# The Effects of Adding Elements of Zinc and Magnesium on Ag-Cu Eutectic Alloy for Warming Acupuncture

**DOI:** 10.1155/2013/532735

**Published:** 2013-08-27

**Authors:** Yu Kyoung Kim, Il Song Park, Keun Sik Kim, Min Ho Lee

**Affiliations:** ^1^Department of Dental Biomaterials, School of Dentistry, Institute of Oral Bioscience, Chonbuk National University, BK 21 Project, Jeonju 561-756, Republic of Korea; ^2^Technical Research Center, Dong Bang Acupuncture, Inc., Boryeong 355-851, Republic of Korea

## Abstract

The warming acupuncture for hyperthermia therapy is made of STS304. However, its needle point cannot be reached to a desirable temperature due to heat loss caused by low thermal conductivity, and the quantification of stimulation condition and the effective standard establishment of warming acupuncture are required as a heat source. Accordingly, in this study, after Ag-Cu alloys with different composition ratios were casted and then mixed with additives to improve their physical and mechanical properties, the thermal conductivity and biocompatibility of the alloy specimens were evaluated for selecting suitable material. Ag-Cu binary alloys and ternary alloys added 5 wt% Zn or 2 wt% Mg were casted and then cold drawn to manufacture needles for acupuncture, and their physical properties, thermal conductivity, and biocompatibility were evaluated for their potential use in warming acupuncture. The results of this study showed that the physical and mechanical properties of the Ag-Cu alloys were improved by additives and that the thermal conductivity, machinability, and biocompatibility of the Ag-Cu alloys were improved by Mg addition.

## 1. Introduction

Warming acupuncture is one of treatment methods that apply physical stimulation to the acupoint in oriental medicine. And it is a kind of combination treatment of acupuncture producing stimulation and moxibustion producing heat stimulation [1]. Moxibustion is a traditional medicine that heats the acupuncture points by burning compressed round-shape moxa (*Artemisia vulgaris*) [2]. Thus, warming acupuncture transmits moxa heat deeply into the body by way of acupuncture points, making circulated the Qi-blood and warmed the meridian system [3]. Warming acupuncture has been recently reported to have an effect on the immune system or osteoarthritis [3, 4], and its effect on hyperhidrosis or weakness diseases has been proven [3, 5].

Gold has superior biocompatibility, which is functionally suitable as needle materials used to warming acupuncture. It has been generally used via various methods such as dietary, application, and bathing. Recently, its use in esthetic procedures has been increasing in oriental medicine or plastic surgery as shown in gold lifting, one of face esthetic therapy, where gold thread is inserted into the wrinkle skin tissue [6]. However, Ag and Au are unsuitable for acupuncture due to their low mechanical strength and elasticity. In addition, Au is clinically reused due to the high price; it causes trouble as medical accidents caused by the damage, in complete cleaning and sterilization of the needle point. So the inexpensive disposable materials are considered as an alternative material replacing the gold alloys.

 Disposable needle materials that are used in general acupuncture, such as stainless 304 or 316 alloy, are currently used for warming acupuncture. Compared to Ag alloy needle with high thermal conductivity, these materials have higher physical strength and low price. Thus, they are used for warming acupuncture as well as general oriental treatments. Lim et al. [7] reported the biostability and cytotoxicity of STS304 needle. Although STS304 needle has superior biocompatibility, it has low thermal conductivity. Thus, as the actual temperature at the insertion site is less than 30°C when STS304 needle is used, it is hard to achieve the effective efficacy of warming acupuncture.

In general, the required temperature of warming acupuncture that is transmitted to the skin tissue for obtaining the effect of warming stimulation is approximately 40~45°C. Froese et al. [8] reported that cytotoxicity was observed when 45°C or higher heat was applied to the nerve root or spinal cord of rats for 10 minutes or longer and that bone tumor tissues were destroyed when they were treated with heat at 65°C for 15~30 min. Thus, if 40~45°C heat is applied to the skin tissue using a material with high thermal conductivity, an effective efficacy could be achieved via warming acupuncture.

Metals have high thermal conductivity in the order of Ag > Cu > Au > Al, among which Ag has a thermal conductivity of 360 Kcal/mhr°C. Among Ag, Cu, and Al that are relatively cheaper than Au, Al is unsuitable as a biomaterial as it is environmentally unfriendly and has neurologic toxicity [9]. Ag and Cu have high antibacterial effect and thermal conductivity. However, Ag and Cu have low abrasiveness due to low hardness when used as pure metals. Furthermore, Ag and Cu alloying processes are required for acupuncture due to low elasticity and tensile strength of pure Ag and Cu. Among elements that are added to increase the physical properties of Ag-Cu alloys, Zn can be solidified inside of Cu phase to achieve solid solution hardening, thereby increasing strength and anticorrosion [10]. Mg is secondly abundant in cells to follow potassium [11] and improves mechanical properties via precipitation hardening in which Mg is partially precipitated after forming an intermetallic compound with other elements [12]. Accordingly, in this study, Ag-Cu binary alloys and Zn or Mg added ternary alloys were casted and then cold drawn to manufacture needles for acupuncture, and their mechanical properties, thermal conductivity, and biocompatibility were evaluated for their potential use in warming acupuncture. 

## 2. Materials and Methods

### 2.1. Sample Preparation

**Figure 1:**
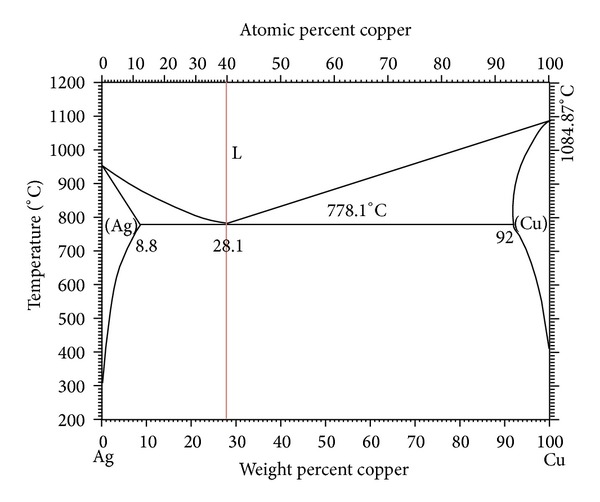
Binary phase diagram of the Ag-Cu system taken from [13].

To determine the basic composition of alloy materials used for warming acupuncture, the composition ratio of Ag-Cu binary alloys was set via the phase diagram [13] shown in Figure 1, of which 5 wt% Zn or 2 wt% Mg was added to Ag-Cu base alloy to prepare ternary alloys (Table 1). Rod type alloys with diameter 5.5 mm and length 100 mm were casted using a high frequency casting machine (Linn, Germany) under Ar condition. To manufacture acupuncture needles, the casted rod type alloys underwent cold drawing. Drawing is a process that is performed to achieve desirable diameter by decreasing cross-section by drawing a material via a die with a leaned hole [14]. The rod was multidrawn under conditions of cross-section reduction rate 15%, die angle 12°, and drawing rate 10 m/min for each pass to prepare a 0.4 mm sized wire. Among the groups listed in Table 1, two binary and two ternary alloys, except for those that cannot manufacture a wire, were selected as specimens of this study. For straightening wire, the wire passed through 3 mm diameter bent tube which rotated at a speed of 7000 rpm. As for the evaluation of wire straightness, after straightening process, the specimens were cut into 100 mm length pieces, and the gap length was measured by setting the central line. 

Phosphor bronze alloy that is commercially available, Ti-6Al-4V alloy that is widely used as a biomaterial, and STS304 that is currently used for warming acupuncture were used as control groups. The detailed composition of the control groups are presented in Table 2. 

### 2.2. Mechanical Analysis

#### 2.2.1. Surface Analysis

The chemical composition on the surface layer was examined by EDX (Oxford, UK) in SEM (JSM-5900, JEOL, Japan).

#### 2.2.2. Measurement of Vickers Hardness

To compare the hardness of the specimens, the wire of each specimen was cut and then mounted using a dental casting investment (Ortho Jet Powder, Lang dental Manufacturing Co., Inc., USA). The surface of the specimens was sequentially polished using number 400~number 2000 SiC papers and then rinsed. The cross-section of the specimens was indented at an indentation load of 300 gf for 10 sec and then measured using a Micro Vickers Hardness Tester (MMT7, Matsuzawa, Japan) seven times. After removing the minimum and maximum values, the mean and standard deviation were calculated using the remaining values. Statistical significance was analyzed using one-way ANOVA test for all measurements. *P* value < 0.05 was considered significant.

#### 2.2.3. Measurement of Tensile Strength

Tensile strength was measured 10 times at a constant crosshead speed of 0.5 mm/s with a 50 mm gauge length using a tensile strength tester (Instron, 4478, USA) with 500 N load cell. Spool type jig has been taken in order to insure an alignment of the wire on the machine axis and broken location. After removing minimum and maximum values, the mean and standard deviation were calculated using the remaining values. 

#### 2.2.4. Evaluation of Anticorrosion

For the evaluation of the anticorrosive characteristics of each specimen, corrosion potential and corrosion current density were measured in 500 mL saline solution using Potentiostatic/Galvanostatic 263A (AMETEK, EG&G/PAR, USA) according to the potentiodynamic polarization. Ag/AgCl electrode and platinum electrode were used as the reference electrode and counter electrode, respectively, and the specimen was connected to the working electrode, followed by measurement in a condition of scanning rate, 3 mV·s^−1^ and RT.

#### 2.2.5. Calculation of Thermal Conductivity

Electrical resistance was measured after applying the current within a certain distance. Each wire was cut into 300 mm sized specimen, and then electrical conductivity (*σ*) was measured using the resistance that was obtained by applying 10 mA current to the both ends of the specimen. Thermal conductivity (*κ*) was calculated using the Wiedemann-Franz-Lorenz law [15].

Theoretically, the proportionality constant *L* is as follows:

(1) κσT=CWFL(CWFL=π2κ23e2=2.44×10−8 W Ω K−2),                    κ=σTCWFL,

where *T* is temperature; wire length is 300 mm; *κ* is Thermal conductivity and *σ* is the electrical conductivity.

### 2.3. In Vitro Test

Osteoblast cell MC3T3-E1 was purchased from ATCC (American Type Culture Collection). Fetal bovine (FBS, Gibco Co., USA), penicillin (Gibco Co., USA), and streptomycin (Gibco Co., USA) were added to *α*-MEM (Gibco Co., USA) to prepare the culture medium. Incubation was carried out at 37°C in an atmosphere containing 5 vol.% CO_2_. According to ISO 10993-5:1999 [16], elution was carried out in an extraction medium with a ratio of *α*-MEM medium : sample volume = 9 : 1 under the condition of a humidified atmosphere with 5% CO_2_ at 37°C for 72 hours. *α*-MEM medium was used as the negative control. 

The cells were incubated in cell culture plates at 2.5 × 10^4^ cells mL^−1^ and incubated for 24 hours to allow cell attachment. The medium was replaced with a 200 *μ*L extraction medium per sample. After incubation in a humidified atmosphere for 2 days, the sample was stained with 0.3% Crystal violet for observations of the cell morphology and was then observed by stereoscopic microscope (DE/EZ4, Leica, Japan). The 3-(4, 5-dimethylthiazol-2-yl)-2, 5-diphenyltetrazolium bromide (Sigma) MTT mixture solution was added to each well and incubated for 4 hours. The medium was removed, formazan solution (DMSO) was added to each well, and the optical density (OD) measurements were conducted at a wavelength of 570 nm using a spectrophotometer. The cell relative growth rate (RGR) was calculated using the following formula:

(2) $$\begin{array}{c} \text{RGR}=\frac{{\text{OD}}_{\text{test}}}{{\text{OD}}_{\text{negative}}}\times 100\%. \end{array}$$

### 2.4. Manufacture of Needles and Analysis of Their Surface Morphology

For the manufacture of needles for warming acupuncture, the wire of each specimen was cut into pieces with a certain length, and then the one end was bent to connect to the handle. After connecting the handle and body, the needle point was grinded using a rotating grinder and then ethanol cleaning, followed by drying. The morphology of the grinded needle point was observed using a SEM (JSM-5900, JEOL, Japan). 

## 3. Results

For the development of a disposable needle suitable for warming acupuncture, Ag-Cu binary alloys added Zn and Mg for ternary alloys were casted and then drawn. 

Ag-Cu binary alloys with different composition ratios were prepared and then drawn to have a diameter of 0.4 mm, followed by assessing their straightness (Table 1). The alloys with Ag 70 and 30 wt% were drawn to have a diameter of 0.4 mm, but the other specimens were cracked and snapped during drawing due to low ductility. Among the binary alloys, the Ag30-Cu70 alloy showed lowest gap length. The definition and composition of the Ag30-Cu70 binary alloy that underwent drawing and straightness process, ternary alloys where Zn or Mg was added to the basic composition of the Ag70-Cu30 alloy, and the control groups, such as phosphor bronze alloy, Ti-6Al-4V alloy, and STS304.

The results of composition analysis are presented in Table 2. In Group a, Zn 5 wt% was added to the Ag-Cu alloy with the near eutectic composition. In Group b, the 70Cu-30Ag alloy with Cu and Ag ratio, which is opposite to Group a, was prepared to form solid solution. In Group c, Mg 2 wt% was added to the eutectic composition of the Ag-Cu alloy instead of Zn in order to improve mechanical strength. In Group d, phosphor bronze used for electric devices, spring, fuse, and measuring equipments, is a Cu-P-Sn ternary alloy. Phosphor bronze was selected as a control group in this study as it has high thermal conductivity, strength, and elasticity. Pure Ti has high biocompatibility as an implantable biomaterial. However, the Ti-6Al-4V alloy (Group e) was selected instead of pure Ti due to its low hardness and elasticity that are unsuitable for acupuncture. Group f is STS304, which is the most commonly used as a disposable acupuncture material. STS304 is comprised of iron as a main composite and added C, Si, and Mn, as minor composites. 

**Figure 2:**
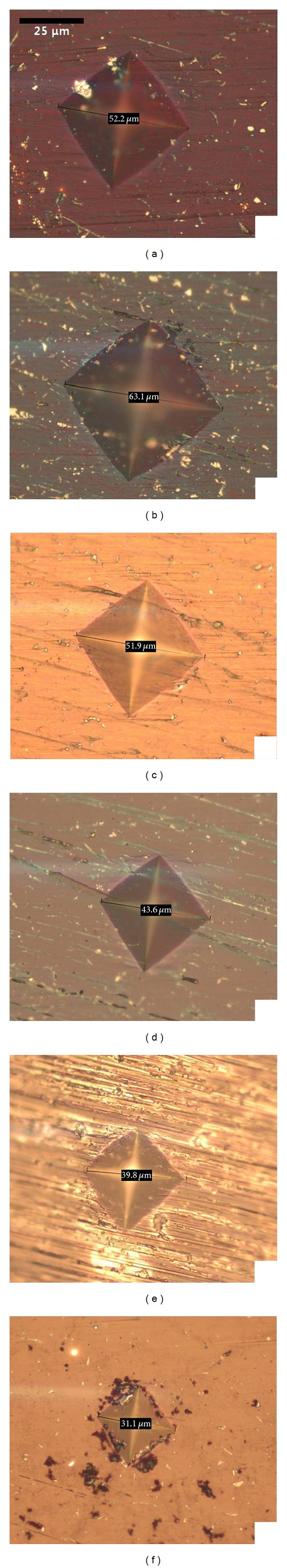
Optical images of Vickers hardness: (a) Ag65-Cu30-Zn5, (b) Cu70-Ag30, (c) Ag68-Cu30-Mg2, (d) phosphor bronze, (e) Ti-6Al-4V alloy, and (f) STS304.

**Figure 3:**
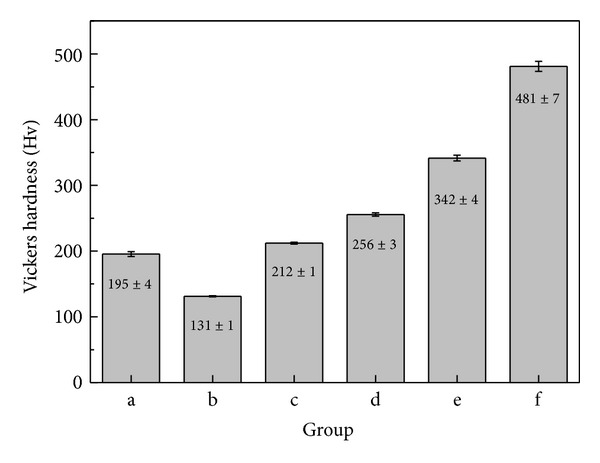
Vickers hardness value of each group: (a) Ag65-Cu30-Zn5, (b) Cu70-Ag30, (c) Ag68-Cu30-Mg2, (d) phosphor bronze, (e) Ti-6Al-4V alloy, and (f) STS304.

The surface images after measuring Vickers hardness showed unevenness that was formed by pressing the indented diamond hexagonal particles onto the specimens (Figure 2). As the hardness of the specimens decreased, the diagonal length of the indented plane increased as shown in Figure 2(b). The results of measuring Vickers hardness are presented in Figure 3. The highest hardness was observed in Group f (STS304) currently used for acupuncture. The lowest hardness was shown to be 131 Hv in Group b, the Cu-Ag binary alloy with no additive. The hardness was shown to be approximately 212 Hv in Group c, the Zn or Mg added Ag-Cu alloy. Thus, a significant difference in the hardness was found among the groups. Among the specimens developed in this study, the highest Vickers hardness was observed in Group c. 

**Figure 4:**
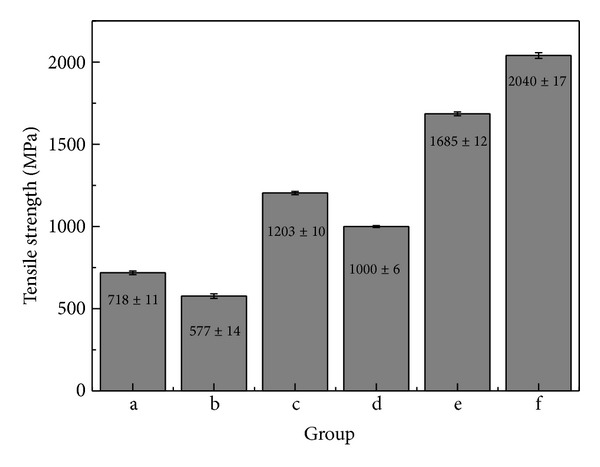
Tensile strength value of each group: (a) Ag65-Cu30-Zn5, (b) Cu70-Ag30, (c) Ag68-Cu30-Mg2, (d) phosphor bronze, (e) Ti-6Al-4V alloy, and (f) STS304.

The results of measuring the tensile strength of the wires are presented in Figure 4. The Groups a and b had low tensile strength under 720 MPa. However, Group c showed higher than commercial alloy (Group d). The tensile strength was significantly improved due to Mg addition. The Ti-6Al-4V alloy as a biomaterial and STS304 which is currently used an acupuncture material were shown over 1600 MPa. And also the electrical resistance and the tensile strength depend on cold working.

 Potentiodynamic polarization was conducted in saline solution to assess anti-corrosion. As a result, the corrosion potential was shown to be −0.289 V in the Ag65-Cu30-Zn5, which was lower than that of Groups b and c developed in this study (−0.248 V and −0.24 V). Meanwhile, the corrosion potential was the highest, but the corrosion current density was low in Group c, the Mg added Ag-Cu alloy. The corrosion potential of Group d was similar with the Ag-Cu alloys. STS304 had the corrosion potential, which was similar to that of Ag alloys and Cu alloys, but Ti-6Al-4V had the lowest corrosion potential. Compared to the other groups, however, STS304 and Ti-6Al-4V had lower corrosion current density and higher anti-corrosion by rapidly forming the passive film. 

The results of measuring electrical resistance are presented in Figure 6. The resistance was measured when 10 mA current was applied to the both ends of the 300 mm length wire. The resistance was shown the highest in Group e. The resistance of Group c, the Mg added Ag-Cu alloy, was significantly lower than that of Group a, the Zn added Ag-Cu alloy. Groups b and d, the Cu alloys, also had low resistance. Based on the principle that resistance is adversely proportional to thermal conductivity or electrical conductivity, the electrical conductivity was calculated and then converted into the thermal conductivity using Wiedemann-Franz-Lorenz law that electrical conductivity is proportional to thermal conductivity (Figure 7). The thermal conductivity was shown to be 499.1 W/mK in the Ag68-Cu30-Mg2 alloy, which was 51-fold higher than that of STS304 and 7-fold higher than that of the Ag65-Cu30-Zn5 alloy.

In this study, staining and MTT test were conducted using MC3T3-E1 from rat osteoblast cells. The 100 mm wires were deposited in the cell medium for 72 hours, and then the eluted solution put to the cells. After 2-day culture, the morphology and survival of the live cells were assessed.

Figure 8 shows the morphology of the live cells. Compared to the cell activity of the negative control (Figure 8(s)), the similar cell activity was observed in Groups e and f, the commercially available alloys. The cell survival decreased in Groups a and c, the Ag alloys. However, the cell membrane activity of Groups a and c was similar to that of the negative control, of which the cell survival was higher in Group C than in Group a. Groups b and d, the Cu alloys, had cytotoxicity, and phosphor bronze, in particular, had the highest cytotoxicity as no stained cell nucleus was observed. This result was consistent with that of MTT test (Figure 9). Given that the cell survival of the negative control was set as 100%, the cell survival was shown to be 85% or higher in Groups e and f, but 50% or lower in Groups b and d, the Cu-based alloys. Among the developed alloys, the cell survival of Group c was shown to be 73.61 ± 0.5%, which was the highest. As this result showed that the biocompatibility of Ag-Cu-X (Zn, Mg) ternary alloy was superior to that of the Cu alloys, needles were manufactured using Groups a and c, and the abrasive state of the needle point was then observed using a SEM (Figure 10). When the needle point was grinded at the same speed of floatstone, the Ag65-Cu30-Zn5 alloy was grinded at the similar grinding angle compared to the commercially available STS304 needle (Figure 10(c)). In the case of the Ag68-Cu30-Mg2 alloy (Figure 10(b)), the grinding angle was narrow, and it was bilaterally symmetrical with the needle point on the center and had smooth surface. When the needle point was magnified and then observed (Figure 10(d)), the Ag65-Cu30-Zn5 alloy had poor machinability as the needle point was not grinded sharply due to its brittleness.

## 4. Discussion

Warming acupuncture is effective in the treatment of neuralgia and arthritis by conducting acupuncture and moxibustion simultaneously, typical treatment options in oriental medicine. In warming acupuncture that consists of acupuncture and moxibustion. The acupuncture stimulates the acupoint physically and the warming effect of moxibustion, which is far-infrared effect. It improves Qi-blood circulation and vitality [1]. In acupuncture, materials with high electrical conductivity should be used to better transmit the heat of moxibustion. However, as pure Au or Ag needle is unsuitable for acupuncture due to its low hardness, tensile strength, and high price, stainless steel needle has been mainly used as a disposable needle for acupuncture in the modern society. The needle is inserted to the body for a certain period of time. Thus, its biostability as well as mechanical properties, such as tensile strength, elasticity rate, wear resistance, and fatigue strength, which is required for manufacturing, should be validated [17]. Several studies reported the thermal characteristics of warming acupuncture using STS304. However, the quantification of stimulation condition and the establishment of effective standard warming acupuncture are required as the temperature of the needle point cannot be reached to a desirable point due to heat loss caused by low thermal conductivity; the accuracy of the results of temperature measurement using moxibustion is low; and temperature difference is significant depending on the position of the needle body [18]. Accordingly, in this study, after Ag-Cu alloys with different composition ratios were casted and then mixed with additives to improve their physical and mechanical properties, the thermal conductivity and biocompatibility of the alloy specimens were evaluated. 

The casted ingot was processed via cold drawing to machined wires with required diameter sizes. Wires processed via cold drawing have advantages of controlling their size accurately and having smooth surface. Furthermore, their mechanical properties can be improved as the strength and hardness due to hardening process, although ductility decreases [19]. Thus, for the selection of the composition of Ag-Cu binary alloys, seven groups were casted by changing eutectic composition and Ag-Cu composition ratio and then cold drawn. As a result, the binary alloys with Ag 72 and 60 wt% were snapped during the drawing due to low ductility. In addition, the binary alloys with Ag 40, 20 wt% (Table 1 (e, g)) was impossible for the specimens with a diameter of 0.8 mm or less. In other words, without metal addictives of binary alloys with Ag-Cu alloy, cold drawing was difficult to be required diameter wire. In general, disposable needles for acupunctures with a diameter of 0.18~0.4 mm are the most frequently used according to acting point and purpose. 

In the case of warming acupuncture or burning acupuncture, needles with a diameter of 0.6 mm or larger may be used for heat transmission, but their use is limited due to significant pain. In this study, the specimens underwent straightness to manufacture wires with a diameter of 0.4 mm or less. 

Wire straightness is performed for the automation of product production, standardization of size, and the improvement of mechanical properties. However, the most significant purpose of wire straightness is to reduce pain upon needle insertion. The straightened needle body minimizes pain during insertion and increases insert power. Wire straightness removes bending and irregularity caused by residual stress remaining in the wires, thereby improving tensile strength, hardness, and elasticity [20]. In this study, drawn binary alloys to diameter of 0.4 mm were Groups c and f. However, after straightness processing, only the Cu-Ag 30 wt% alloy satisfied the gap length less than 3 mm referred by the central line. The Ag-Cu 30 wt% alloy was drawn to a desirable diameter, but it is required to alloying with Zn and Mg to improve straightness, elasticity, and strength. The Cu amount was fixed, and then the Ag amount was changed according to the addition of Zn or Mg. Usually Zn is increased strength via solid solution hardening effect when it solidified on Cu-rich phase [21]. Magnesium is an essential mineral in the body, and it is used as a biomaterial due to its high biocompatibility and specific strength [22]. In addition, Mg increases electrical conductivity and softening temperature when added to Cu-Ag-P alloy [12].

**Figure 5:**
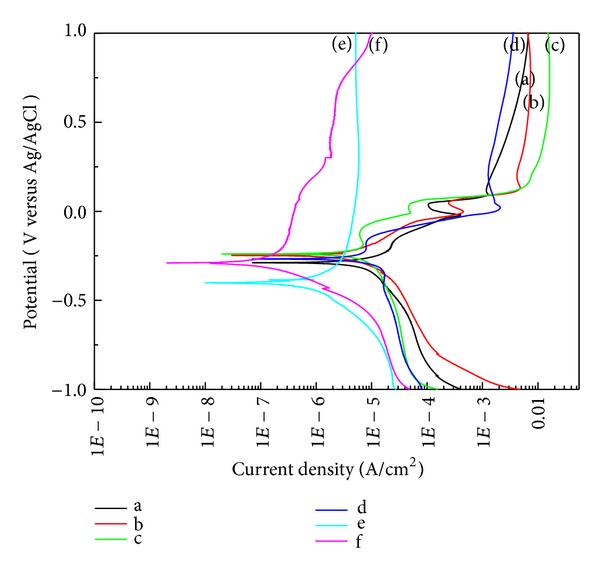
Potentiodynamic polarization curve obtained in a saline solution: (a) Ag65-Cu30-Zn5, (b) Cu70-Ag30, (c) Ag68-Cu30-Mg2, (d) phosphor bronze, (e) Ti-6Al-4V alloy, and (f) STS304.

**Figure 6:**
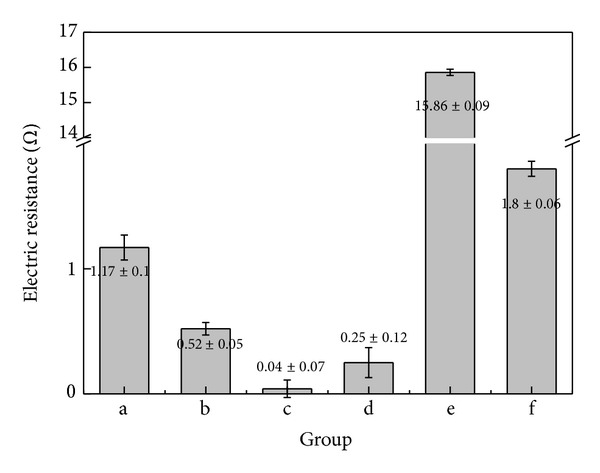
Electric resistance value of (a) Ag65-Cu30-Zn5, (b) Cu70-Ag30, (c) Ag68-Cu30-Mg2, (d) phosphor bronze, (e) Ti-6Al-4V alloy, and (f) STS304.

**Figure 7:**
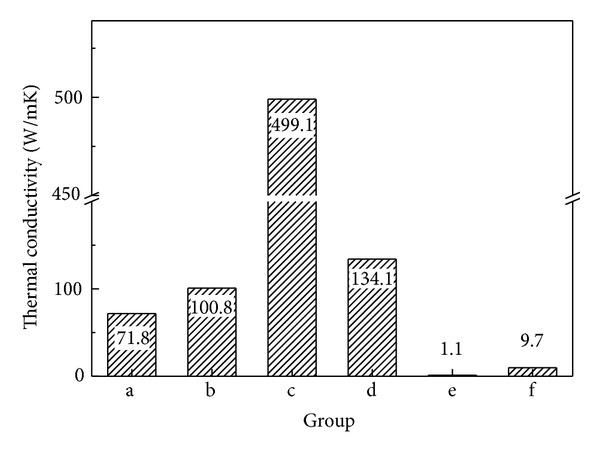
Converted to thermal conductivity from electric resistance value: (a) Ag65-Cu30-Zn5, (b) Cu70-Ag30, (c) Ag68-Cu30-Mg2, (d) phosphor bronze, (e) Ti-6Al-4V alloy, and (f) STS304.

**Figure 8:**
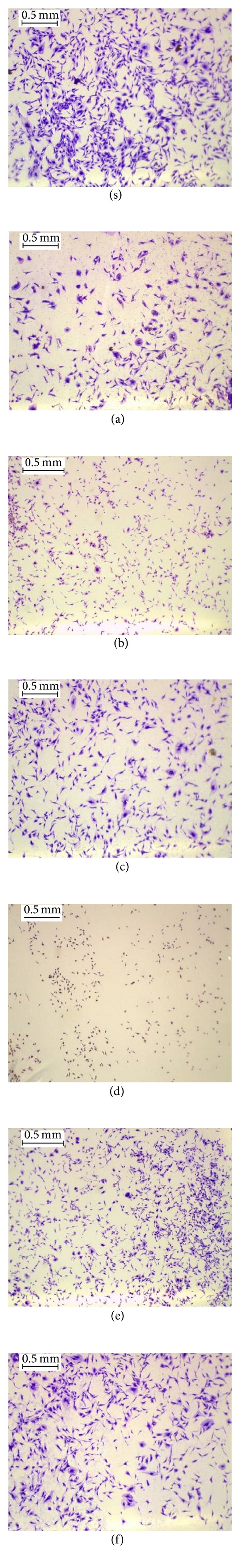
MC3T3-E1 cell morphology for 2 days by elution (s) standard (negative control): (a) Ag65-Cu30-Zn5, (b) Cu70-Ag30, (c) Ag68-Cu30-Mg2, (d) phosphor bronze, (e) Ti-6Al-4V alloy, and (f) STS304.

**Figure 9:**
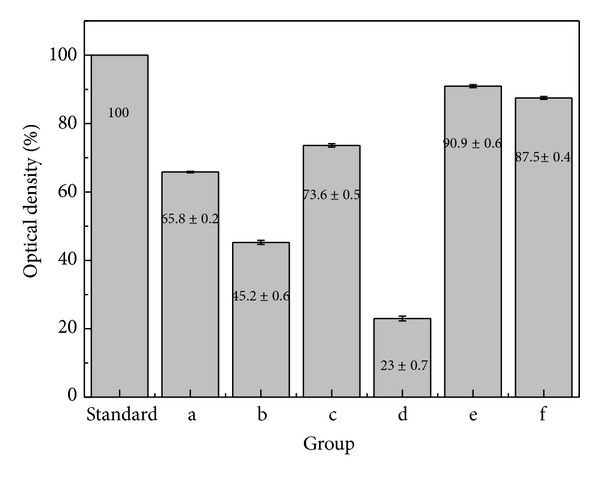
Percentage of optical density for 2 days by MTT test (s) standard (negative group): (a) Ag65-Cu30-Zn5, (b) Cu70-Ag30, (c) Ag68-Cu30-Mg2, (d) phosphor bronze, (e) Ti-6Al-4V alloy, and (f) STS304.

**Figure 10:**
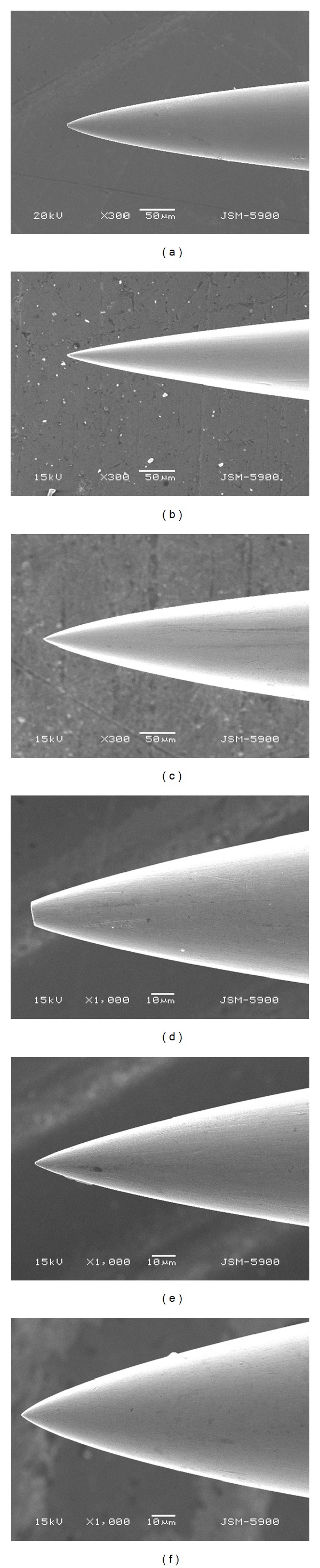
SEM image of machined needle tips: (a) Ag65-Cu30-Zn5 (×300), (b) Ag68-Cu30-Mg2 (×300), (c) STS304 (×300), (d) Ag65-Cu30-Zn5 (×1000), (e) Ag68-Cu30-Mg2 (×1000), and (f) STS304 (×1000).

The result of measuring Vickers hardness showed that the hardness increased by 70 Hv or more in the Zn or Mg added Ag-Cu alloy with the eutectic composition compared to Group b (Figures 2 and 3). Eutectic alloy has fine grain structure, thereby achieving superior mechanical properties such as tensile strength and hardness [23]. However, as a Vickers hardness of approximately 200 Hv or higher is required for needles used in acupuncture, the Mg 2 wt% added alloy had suitable hardness for acupuncture. In addition, compared to phosphor bronze, Group c had lower hardness, but its tensile strength increased by 200 MPa or higher. If a small amount of Mg is added to Ag alloys for improving hardness. It is attributable to dispersion hardening [24]. In the connection of the needle handle and body, the minimum tensile strength was shown to be 1000 N/mm^2^. Only Group c had the tensile strength that satisfied the reference value. In the potentiodynamic polarization curve that was conducted for anti-corrosion evaluation, no significant difference in the current density and corrosion potential was found among the three groups of Cu-Ag alloys series and phosphor bronze. The corrosion potential of the aforementioned specimens was higher than or similar to that of STS304 that is currently used as a disposable needle. However, due to the high corrosion current density of three groups of Cu-Ag alloys series and phosphor bronze, their anti-corrosion was relatively lower than that of STS304. Group e had lower corrosion potential than that of the three groups of Cu-Ag alloys (Figure 5 (a–c)) but had lower corrosion rate due to the formation of passive film.

In general, strength and hardness are adversely proportional to electrical conductivity. However, as Ag-Cu alloy systems are eutectic alloys including incomplete solid solutions, they have superior flowability, castability, and electrical conductivity at the near eutectic composition [25]. When elements are added to obtain superior mechanical properties, electrical conductivity is rapidly changed according to element addition. This is attributable to the thermal oscillation of the crystal lattice, other types of atoms or ions brought as impurities, and lattice defects. These factors, alone or in combination, decrease the electrical conductivity of alloys [26]. However, in the measurement of electrical resistance, the electrical resistance was shown to be 0.04 Ω in Group c, which was the lowest. Meanwhile, the electrical resistance was shown to be 1.17 Ω in Group b, the Zn added alloy, which was the highest among the Ag-Cu alloys. In addition, the conductivity was shown to have decreased in Group b as the formation of the secondary phase such as Cu_2_O reduced the volume of Cu that can transmit the current. 

According to the Wiedemann-Franz law [27], as the ratio of thermal conductivity (*κ*) to electrical conductivity (*σ*) is constant, electrical conductivity can be converted into thermal conductivity using Lorenz constant *L* = 2.44 × 10^−8^ W Ω K^−2^. To obtain alloys that have superior strength while minimizing thermal conductivity reduction, it is required an element for addition, which has low solid degree for a base metal and can be precipitated as an intermetallic compound. In this study, the thermal conductivity of added Mg was shown to be 499.1 W/mK, which was the highest. It was sevenfold higher than that of the Zn added alloy, which showed that the thermal conductivity varied depending on additives. In particular, the thermal conductivity was 50-fold higher in the alloy that had Mg addition than in STS304 that has been currently used for warming acupuncture therapy. Added solute atoms with various elements cause local change of elasticity, during which electrons are dispersed as the lattice periodicity of the base alloy is intervened. Thus, the additions of Zn to alloys affect electrical conductivity and thermal conductivity [28].

In this result, Mg element addition to alloys was to increase the thermal conductivity of the alloys suitable for warming acupuncture. However, high biocompatibility is also required as needles are inserted to the body. The anti-corrosion of Groups b and d, the Cu alloys, was similar to that of the Ag alloys. However, the cell activity of the Cu alloys was shown to be 50% or lower, and the cell survival of Group d was particularly lower (22.97%). Although Cu metal and eluted Cu have high bacterial resistance [29], it is difficult to use Cu materials as biomaterials due to high cytotoxicity. Ag is used as a medical and biomaterial because of its potent antibacterial effect and biocompatibility [30]. Ag^+^ ion has a potent antibacterial effect against various types of bacteria at a lower concentration. In Group c (added Mg) the cell survival was higher than Group a added Zn. MgO formed in the alloy has high hardness, thereby increasing the strength and interatomic bonding force of alloys [31], which results in the improvement of cell survival by reducing pH change caused by the eluted alloy elements, impurities, and adjacent body fluid. 

In Groups a and c, the alloys developed in this study, their anti-corrosion and mechanical properties were similar each other. However, the thermal conductivity and biocompatibility were improved after Mg addition. In addition, the sharpness of the needle point during needle manufacturing is determined by hardness, strength, and elasticity. When grinded with the same force, machinability was lower in Group a than in Group c. The results of this study showed that the physical and mechanical properties of the Ag-Cu alloys were improved by additives, and that the thermal conductivity, machinability and biocompatibility of the Ag-Cu alloys were improved by Mg addition. 

## 5. Conclusion

In the development of a new alloy for warming acupuncture, ternary alloys were developed by adding Zn or Mg to the Ag-Cu alloys with eutectic composition, and binary alloys were developed based on Cu metal. The physical and mechanical properties, thermal conductivity, and biocompatibility of the developed alloys were assessed, and the following results were obtained.

- Zn or Mg was added to the casted Ag70-Cu30 alloy at the fixed Cu content, and then the Zn or Mg added alloys were cold drawn to prepare wires as 0.4 mm diameter.
- After Zn or Mg addition, the hardness and tensile strength of the alloys were improved, but no significant change in the anti-corrosion was found.
- After Mg addition, the tensile strength, thermal conductivity, and biocompatibility of the alloy were more improved compared to the Ag65-Cu30-Zn5 alloy. In addition, the narrowed grinding angle in the grinding of the needle point was expected to contribute to reduce resistance upon needle insertion into the body.

In conclusion, compared to the Cu-Ag alloys, the Ag-Cu alloys had superior physical and mechanical properties and biocompatibility. In particular, the tensile strength and anti-corrosion of the Ag-Cu-Zn alloy were insignificantly improved after Zn addition. Meanwhile, Mg addition could be useful in the application of the Ag-Cu-Mg alloy to needle materials for warming acupuncture as it provides high thermal conductivity and biocompatibility.

## Figures and Tables

**Table 1:** Results of composition, drawing diameter, and straightness.

Materials	Ag (wt%)	Cu (wt%)	Zn (wt%)	Mg (wt%)	Drawing process (diameter, mm)	Straightness (gap length, mm)
Ag80	80	20			0.5	Broken wire
Ag72	72	28			3	Broken wire
Ag70	70	30			0.4	3.85 ± 0.41
Ag60	60	40			4.5	Broken wire
Ag40	40	60			0.7	Broken wire
Ag30	30	70			0.4	2.42 ± 0.31
Ag20	20	80			0.8	Broken wire
Ag65-Cu30-Zn5	65	30	5		0.4	2.94 ± 0.81
Ag68-Cu30-Mg2	68	30		2	0.4	2.12 ± 0.35

**Table 2:** Chemical composition of alloys (a) Ag65-Cu30-Zn5, (b) Cu70-Ag30, (c) Ag68-Cu30-Mg2, (d) phosphor bronze, (e) Ti-6Al-4V alloy, and (f) STS304.

Group	Cu	Zn	Ag	Mg	Sn	Al	Ti	V	C	Si	Cr	Mn	Fe	Ni
a	27.41	4.93	67.65											
b	66.94		33.06											
c	29.14		68.49	2.14										
d	93.39				6.61									
e						6.25	89.86	3.88						
f									4.08	0.70	18.83	1.26	67.08	8.04
